# Gender-based differences in brain and plasma pharmacokinetics of letrozole in sprague-dawley rats: Application of physiologically-based pharmacokinetic modeling to gain quantitative insights

**DOI:** 10.1371/journal.pone.0248579

**Published:** 2021-04-02

**Authors:** Priyanka Arora, Gary Gudelsky, Pankaj B. Desai

**Affiliations:** Division of Pharmaceutical Sciences, James L. Winkle College of Pharmacy, University of Cincinnati, Cincinnati, Ohio, United States of America; Monash University, AUSTRALIA

## Abstract

Based on the discovery that the estrogen synthase aromatase (CYP19A1) is abundantly expressed in high- grade gliomas, the aromatase inhibitor, letrozole is being investigated in pre-clinical models as a novel agent against this malignancy. Here, we investigated the systemic and brain pharmacokinetics of letrozole following single and steady state dosing in both male and female Sprague-Dawley rats. Furthermore, we employed physiologically-based pharmacokinetic (PBPK) modeling to gain quantitative insights into the blood-brain barrier penetration of this drug. Letrozole (4 mg/kg) was administered intraperitoneally daily for 5 days (for males) and 11 days (for females) and intracerebral microdialysis was performed for brain extracellular fluid (ECF) collection simultaneously with venous blood sampling. Drug levels were measured using HPLC and non-compartmental analysis was conducted employing WinNonlin®. Simcyp animal simulator was used for conducting bottom-up PBPK approach incorporating the specified multi-compartment brain model. Overall, marked gender-specific differences in the systemic and brain pharmacokinetics of letrozole were observed. Letrozole clearance was much slower in female rats resulting in markedly higher plasma and brain drug concentrations. At steady state, the plasma AUC 0–24 was 103.0 and 24.8 μg*h/ml and brain ECF AUC 0–12 was 24.0 and 4.8 μg*h/ml in female and male rats, respectively. The PBPK model simulated brain concentration profiles were in close agreement with the observed profiles. While gender-specific differences in letrozole PK are not observed in the clinical setting, these findings will guide the dose optimization during pre-clinical investigations of this compound. The PBPK model will serve as an important clinical translational tool.

## Introduction

High-grade gliomas (HGGs) remain one of the most intractable neoplastic diseases with a median survival ranging from only 1 to 4.7 years [1, 2]. Over the years, the standard of therapy for HGGs has essentially remained the same, which includes maximal safe tumor resection upon diagnosis, followed by adjuvant radiation and chemotherapy [3, 4]. In that regard, temozolomide (TMZ), a DNA alkylating agent approved more than a decade ago, is the only FDA-approved drug currently utilized. Unfortunately, the disease recurs in almost all patients [5] with few, if any, therapeutic options available thereafter. Therefore, there remains an urgent need for novel “druggable” targets for HGG treatment. Major impediments for limited therapeutic options include lack of validated targets and limited access of drugs across the blood-brain barrier (BBB) [6].

To that end, novel findings from our laboratory indicate that HGGs overexpress aromatase (estrogen synthase; CYP19A1) enzyme [7] and that letrozole, an aromatase inhibitor (AI), may have potent efficacy for treating HGGs [8]. Moreover, aromatase expression has been shown to be a prognostic biomarker for astrocytoma patients [9]. Consequently, preclinical/ clinical investigations on the distribution and penetration of AIs across the BBB is of current interest. Of the various AIs being clinically utilized, letrozole is an ideal candidate to be used as a neurotherapeutic since its physicochemical and pharmacokinetic properties meet the requisite criteria for central nervous system (CNS) penetration [10], viz. small molecular weight (285 Da), lipophilicity (log P ≈ 2.5), low plasma protein binding (≈60%) and turnover, and high oral bioavailability (almost 100%). Additionally, unlike other AIs such as vorozole and anastrozole, letrozole is not a substrate for the efflux transporter P-glycoprotein (P-gp) [11] which has been shown to promote active efflux at the BBB thereby limiting CNS penetration [12]. Employing microdialysis, a commonly used technique for studying drug disposition of anti-cancer compounds [13], in previous studies we have shown that letrozole easily crosses the BBB and accumulates in brain and brain tumor tissue as evidenced by brain-to-plasma partition coefficients ranging from 0.5–1.0 in female Sprague- Dawley rats [14]. Furthermore, letrozole intra-tumoral levels were shown to be 1.5 to 2–fold higher relative to the contralateral tumor-free region [14]. More importantly, employing micro-PET/CT imaging letrozole demonstrated significant *in vivo* efficacy in the C6 orthotopic tumor implantation model in female rats showing a marked reduction in the tumor mass [8].

Collectively, these results suggest that aromatase can be exploited as a novel target for HGGs and that letrozole can be used as a potential therapeutic option either as a primary agent and/or as an adjunct to TMZ. However, further optimization of pre-clinical dosing strategies in various animal models warrants detailed systemic and brain PK studies. Previous studies have reported considerable gender-dependent differences in the rat liver microsomal metabolism [15] and in the systemic pharmacokinetics of letrozole in rats [16]. While such differences are not observed clinically, for further pharmacokinetic-guided pre-clinical studies, such differences need to be clearly delineated to ensure that adequate systemic exposure, and perhaps more importantly, brain exposure is attained for therapeutic efficacy. Our current knowledge of the extent of letrozole accumulation and brain disposition upon chronic dosing in rodents is limited. Indeed, all of our preclinical letrozole PK data thus far have been derived from single dose studies performed in female rats. Thus, the primary aim of this study was to evaluate steady state systemic and brain exposure of letrozole in both male and female rats.

Recent advances in PBPK modeling, especially those designed to enhance our insights regarding the penetration and accumulation of drug in the brain, have greatly aided pre-clinical development and translational research of investigational agents with neurological applications [17–20]. By incorporating drug-specific (i.e., permeability, metabolism, solubility, molecular weight etc.) and physiological parameters (e.g., brain volume, composition etc.) into a quantitative framework, PBPK models can be used for enhancing our understanding of the extrinsic (dose, formulation and route of administration) and intrinsic (physiological factors and the resultant drug distribution and clearance) factors and developing correlation between dose and organ-specific drug availability. Ultimately, the derived relationships serve as important basis for translating pre-clinical data and providing important clinically relevant perception. Thus, leveraging our pre-clinical data, we developed a whole body PBPK model integrated with a multi-compartment brain model, as implemented in Simcyp v17, to predict the systemic and brain ECF concentrations of letrozole in rats.

## Materials and methods

### Chemicals and supplies

Letrozole was purchased from Toronto Research Chemicals (Toronto, Canada) and was prepared in bacteriostatic normal saline solution (Braun Medical Inc., Bethlehem, PA) containing 10% Tween-20 (VWR Scientific, Philadelphia, PA) to obtain a final concentration of 2 mg/ml. Methyl tertiary butyl ether, methanol and acetonitrile of HPLC grade were purchased from Fisher Scientific (Hanover Park, IL) while the heparin-dextrose lock solution was obtained from Sai Infusion Technologies (Lake Villa, IL).

Other reagents were of analytical grade and were purchased from Fisher Scientific (Hanover Park, IL). Microdialysis membrane with an outside diameter of 216μm and a molecular weight cut-off of 13 kDa was obtained from Spectrum laboratories (Rancho Dominguez, CA) and K2- EDTA coated tubes were purchased from VWR Scientific (Philadelphia, PA).

### Animals

Age-matched (7–9 weeks) adult female (201–225 g; N = 10) and male (301–325 g; N = 10) jugular vein cannulated Sprague-Dawley rats (Charles River Laboratories) were used for this study. The experiments were conducted in strict accordance with the University of Cincinnati Institutional Animal Care and Use Committee (UC IACUC)-approved protocol (Protocol Number: 10-11-01-01) and all the studies were performed as per the highest international standards of animal welfare outlined by the NIH’s Guide for the Care and Use of Laboratory Animals. Upon arrival, animals were housed individually under pathogen-free, temperature and humidity-controlled environment on a 12/12 h light/dark cycle and were given access to standard chow and water ad libitum (ad-lib). To maintain the patency of the jugular vein catheters, they were flushed with normal saline and locked with the heparin-dextrose solution every other day. As part of monitoring, we ensured that animals were eating and drinking ad-lib, gaining weight while housed in our animal facility and showed no overt neuromotor or other symptoms.

### Construction of microdialysis probes

Concentric-style microdialysis probes with an outer diameter of 210 μm and an active length of 4.5 mm were constructed with hollow fiber membrane (13 kDa molecular weight cut-off). The inlet tubing was PE-20 (0.38 mm I.D., 1.09 mm O.D., 8 cm long, Becton Dickinson, Sparks, MD) and the outlet tubing was fused silica (75 μm I.D., 147 μm O.D., 5 cm long, Polymicro Technologies, Phoenix, AZ) within Tygon tubing (21cm long, Fischer Scientific, Hanover Park, IL). Probe components were affixed to 26G hypodermic tubing (0.01in I.D., 0.018in O.D., 19mm long) using epoxy. The average *in vitro* relative recovery of letrozole was determined at a flow rate of 2.0 μ1/min at 35°C.

### Microdialysis probe implantation surgery

Microdialysis probes were surgically implanted in jugular vein cannulated rats under anesthesia (ketamine/ xylazine 70/ 6 mg/kg i.p., Henry Schein, NY) one day prior to the experiment. Buprenorphine (Buprenex®, Henry Schein, NY) was administered for perioperative analgesia. The probe was inserted into the striatum with the following tip coordinates: A/P, 1.2 mm, L, 3.1 mm, and D/V −7.8 mm, according to the stereotaxic atlas of Paxinos and Watson [21]. The active portion of the membrane for the probes was 4.5 mm. Following implantation, the probes were connected to an infusion pump set to deliver modified Dulbecco’s phosphate buffered saline containing 1.2 mm CaCl2 and 5 mM glucose at a flow rate of 1 μl/min overnight. On the day of the experiment, the flow rate was increased to 2 μl/min and the probes were allowed to equilibrate for 2 hours before dialysate sample collection.

### Drug administration and blood/brain ECF sample collection

#### Single dose

Following probe equilibration, letrozole was administered via the intraperitoneal route at a dose of 4mg/kg. Blood samples were collected in K2- EDTA coated tubes through the jugular vein catheters at the following time points- pre-dose, 1, 2, 4, 6, 8, 12, 14, 20, 24, 36, 48 and 72 h post administration. Within 10 minutes of collection, plasma was separated by centrifugation of the blood samples at 4,000 rpm for 15 min. Microdialysis samples were collected simultaneously through the probes at the same time points from 0–12 hours. All the samples were stored at -80°C until analyzed.

#### Steady state

To establish the steady state, letrozole was administered via the intraperitoneal route at a dose of 4mg/kg o.d for 11 days (female rats) and 5 days (male rats) based on the previously published data and our preliminary experiments. On the day of the experiment (last day of dosing), blood and dialysis samples were collected concurrently as described above at the following time points- pre-dose, 1, 2, 4, 6, 8 and12 h post administration. An additional 24 hr time point was included in the plasma sample collection and all the samples were stored at -80°C until analyzed.

### Sample analysis

#### Sample preparation

Letrozole was extracted from the plasma samples using the liquid-liquid extraction technique. Calibration standards were freshly prepared by spiking working letrozole solution into the blank rat plasma to give a concentration curve ranging from 5 to 5000 ng/ml. Briefly, 50 μl of the plasma sample/standard spiked with known letrozole concentration was transferred to a glass tube containing 1 mL of the extraction solvent methyl-tertiary-butyl ether. All the samples were then vortex mixed for 40 sec and centrifuged for 5 min. 800 μl of the supernatant was decanted into a clean test tube and evaporated to dryness using a CentriVap evaporator. The precipitate was reconstituted in 100 μl of the mobile phase and vortex mixed, following which, 50 μl sample volume was injected onto the HPLC system for analysis. The dialysis samples, on the other hand, were injected directly onto HPLC.

#### Chromatographic condition

The chromatographic analysis was performed under ambient conditions with the Waters HPLC system employing a reversed phase Waters BDS Hypersil C18 column (100 x 4.6 mm, particle size of 5 μm) and a Waters scanning fluorescence detector (Model 474) with excitation and emission wavelengths of 230 and 295 nm, respectively. Mixture of phosphate buffer: acetonitrile (65:35, v/v; pH 10.2) at a flow rate of 1.0 mL/min was used as the mobile phase. Letrozole peaks were detected at a retention time of around 2.6 min. Three sets of calibration plots were constructed for method validation. Linearity was evaluated at 10 standards covering the concentration range of 5–5000 ng/mL and the lower limit of detection was 5 ng/ml. The intra and inter-assay precision for letrozole varied from 0.7–3.9% RSD for the above-mentioned range.

### Pharmacokinetic data analysis

Non-compartmental analysis (NCA) was performed using Phoenix WinNonlin® software (version 6.2.1, Pharsight, St. Louis, MO, USA) and the PK parameters determined included peak concentration (Cmax), time to reach peak concentration (Tmax), area under the concentration-time curve between time zero to the last observed concentration time point (AUC0- t) and the elimination half-life (*t1/2*). Actual sampling times were employed for calculations except for the pre-dose times, which were kept as zero. Since the blood sampling period spanned less than 4.5 half-lives in female rats, plasma AUC0-∞ values could not be determined reliably. Consequently, we were constrained to reporting AUC0-t values for letrozole in this study which was calculated using the linear trapezoidal method. For consistency, only AUC_0-t_ values were reported for male rats as well. Cmax and Tmax values were obtained directly from the concentration-time data. The overall elimination rate constant (*K*10) was determined from the best fitting non-linear regression curve of the terminal portion of the concentration-time profile and *t*1/2 = ln (2)/*k*10. The observed accumulation ratio (Rac) was calculated as:

$$Rac=\frac{Cmax\;or\;AUC(0-12)\;on\;Day\;5\;or\;11}{Cmax\;or\;AUC(0-12)\;on\;Day\;1}$$ (1)

Whilst, the theoretical/ predicted accumulation ratio, Rac’, which is a function of dosing interval (τ) and the half-life was calculated as:

$${Rac}^{\prime }=\frac{1}{1-e^{-k10*\tau }}$$ (2)

Brain ECF concentrations were time-averaged over the collection interval and were corrected for the observed relative recovery. The brain-to-plasma unbound partition coefficients (Kpuu) were calculated as a ratio of the Cmax or AUC0-12 of letrozole levels in the brain ECF to corresponding plasma Cmax and AUC values corrected for plasma protein binding. Employing the ultrafiltration method as described by Lee et. al. [22], our previous study has determined the free unbound fraction of letrozole in plasma to be 38% [14].

### PBPK modeling and simulation

**Fig 1:**
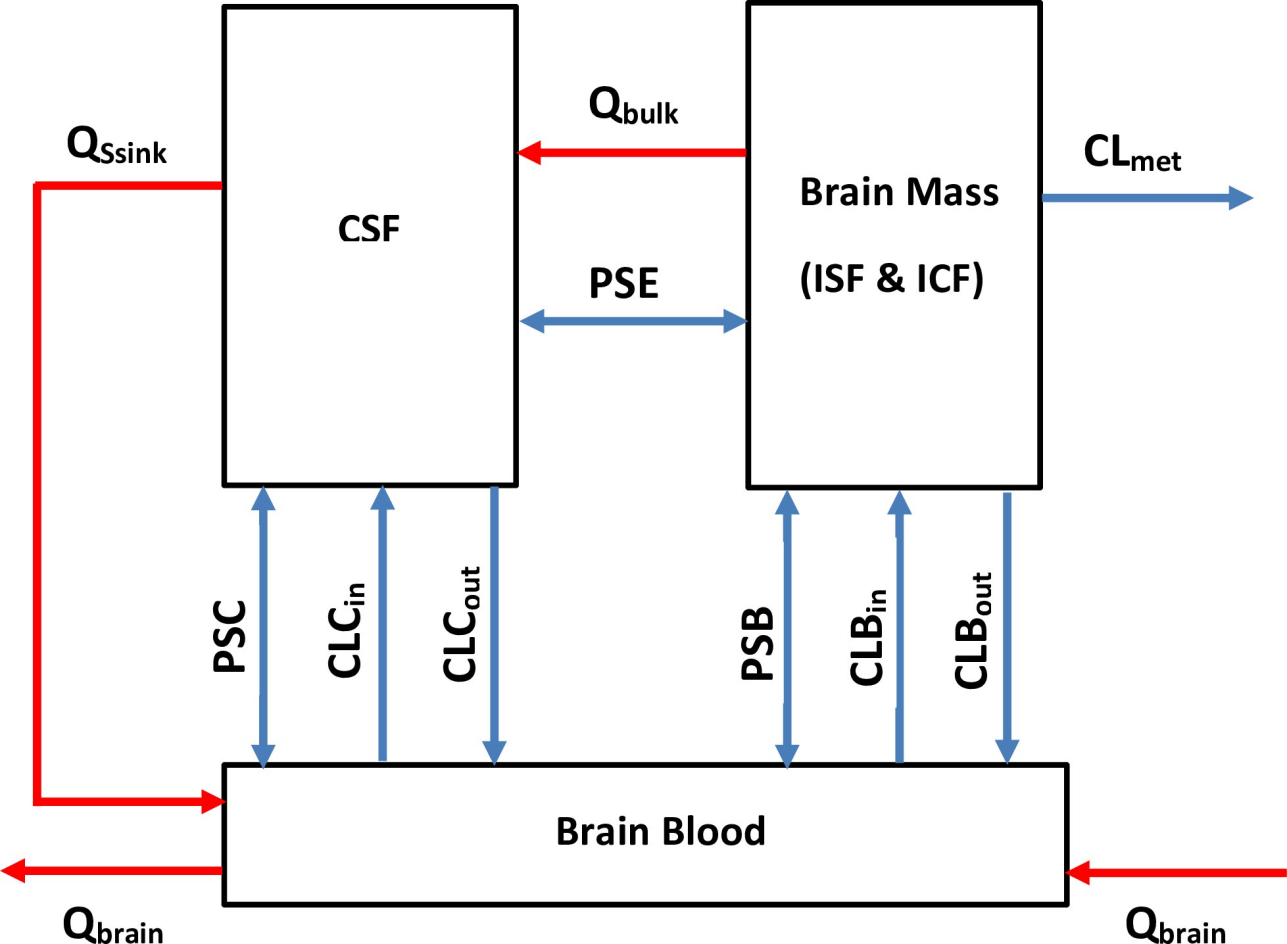
Multi-compartment brain model in rodents as defined in Simcyp Animal Simulator V17. The structure of the multi-compartment brain model in rodents includes three compartments viz., the brain blood, brain mass and the CSF. Qbulk and QSsink represent the bulk flow from the brain mass to the CSF compartment and the CSF flow out of the CSF into the brain blood compartment, respectively while Qbrain is the cerebral blood flow through which the multi-compartment brain model is connected to the whole body. CLBin, CLBout, CLCin and CLCout are the overall clearance of uptake and efflux transporters expressed at BBB and BCSFB respectively and CLmet (L/h) is the metabolic clearance due to brain enzymes. PSB, PSC and PSE denote the passive permeability- surface area products on the BBB, BCSFB and CSFBB, correspondingly.

Simcyp Animal Simulator V17 (Certara Inc.) was employed for developing a bottom-up whole body PBPK model incorporating the specified multi-compartment brain model. Based on its anatomy and physiology, the whole brain is described by three compartments, viz. brain blood, brain mass and cerebrospinal fluid (CSF) as illustrated in Fig 1 (Simcyp® Rat Simulator). For model development, the physiological/ system-specific parameters were inferred from the existing rodent species within the animal simulator while, the key drug- specific parameters used are summarized in Table 1. Briefly, all the physiochemical and binding to blood components (plasma proteins and erythrocytes) parameters of letrozole were derived from expansive literature review. The first-order rate of absorption (Ka) and bioavailability following oral administration and systemic clearance values in male and female rats were obtained from a previously reported pharmacokinetic study [16] and the first-order absorption model best fit the letrozole data. Full-PBPK model was employed for drug distribution while the Rodgers and Rowland method [23, 24] or “Method 2”, as defined in Simcyp v17, predicted the volume of distribution (Vss). For the multi-compartment brain model, the passive permeability-surface area product on the BBB (PSB) was determined using the Crone- Renkin Equation [25, 26]:

$$PS_{B}=-F_{pf}ln\;\left(1-\frac{K_{in}}{F_{pf}}\right)$$ (3)

where, *Fpf* is the cerebral blood or perfusion flow rate and *Kin* is the brain uptake clearance. We used 0.036 mL/sec/g as the *Fpf* for letrozole, based on a previous study that obtained an average cerebral perfusion flow rate value for lipophilic compounds [25]. *In situ* mouse brain perfusion studies revealed that the *Kin* value for letrozole is 0.422 ± 0.068 mL/min/g brain [11]. The passive permeability-surface area product on the blood-cerebrospinal fluid barrier (BCSFB), PSC, was assumed as half of the PSB [19, 27] while the passive permeability-surface area product on the cerebrospinal fluid-brain barrier (CSFBB), PSE, was set at 80 mL/min, assuming a high permeability of this barrier [19, 27]. Brain ECF concentrations were predicted using the *in vivo* unbound fraction in the brain (*fu*, *brain*) for letrozole multiplied by the simulated total brain concentrations. The fu, brain for letrozole was experimentally determined in both healthy and tumor-bearing female Sprague-Dawley rats (N = 4/ group). Following single dose intraperitoneal administration of 4 mg/kg letrozole, both brain ECF (representative of unbound drug concentration in the brain) and whole brain tissue samples were collected at the same corresponding timepoint around the T_max_ (~6 hrs) of letrozole in brain ECF and the fu, brain was calculated based on the ratio of observed brain ECF to the whole brain tissue homogenate concentrations of letrozole (0.58 ± 0.19; mean ± SD).

**Table 1:** Key drug-specific input parameters used for letrozole simulations.

Parameter	Value	Comments/ reference
*Physicochemical*		
MW (g/mol)	285	Chemicalize database
logP	≈ 2.5	Chemicalize database
Blood-to-plasma partition ratio	0.8	CDER NDA 20–726
Fraction unbound in plasma	0.4	CDER NDA 20–726; [14]
***Absorption*: *First-Order Absorption Model***		
Intrinsic solubility (μg/mL)	62	Chemicalize database
Fa	0.99	[16]
Ka (1/h)	0.29 (males); 0.49 (females)	[16]
Qgut (mL/min)	1.135	Simcyp Predicted
***Distribution*: *Full PBPK model***		
Vss (L/kg)	3.29	Predicted by Rodgers and Rowland method (Method 2), Kp scalar of 1
***Elimination*: *In vivo clearance***		
CLpo (mL/min)	0.77^*a*^ (males); 0.21^*a*^ (females)	[16]
***Multi-Compartment Brain Model***		
PSB (mL/min)	0.84^*b*^	Determined using Eq (3)
PSC (mL/min)	0.42	Assumed to be half of PSB [19, 27]
PSE (mL/min)	80	Assumed given the high permeability of this barrier [19]
Fraction unbound in brain	0.58	Experimentally determined from *in vivo* rodent studies

*a*Based on CL = 0.185 (males) and 0.051 (females) L/h/kg, assuming 250 g average body weight.

*b*Based on PSB = 0.469 mL/min/g brain weight, assuming 1800 mg brain weight.

For evaluation of model predictions, the PBPK model developed was used to simulate extravascular administration of single/ multiple doses of letrozole (4 mg/kg) in rodents. To evaluate the predictive performance of the PBPK model, we compared the ratio of the predicted exposure change (Cmax and AUC ratios) against the observed data. A 1.25-fold under‐ or over‐prediction was considered good [28] and within 1.5‐fold was deemed reasonable [29, 30].

### Statistical analysis

Key pharmacokinetic parameters of letrozole in male and female rats were evaluated for statistical differences using the Analysis of Variance method followed by the two-tailed t-test. All data were expressed as the mean ± standard deviation (SD). Differences were considered statistically significant at p-value < 0.05.

## Results

### Single dose pharmacokinetics of letrozole in male versus female rats

**Fig 2:**
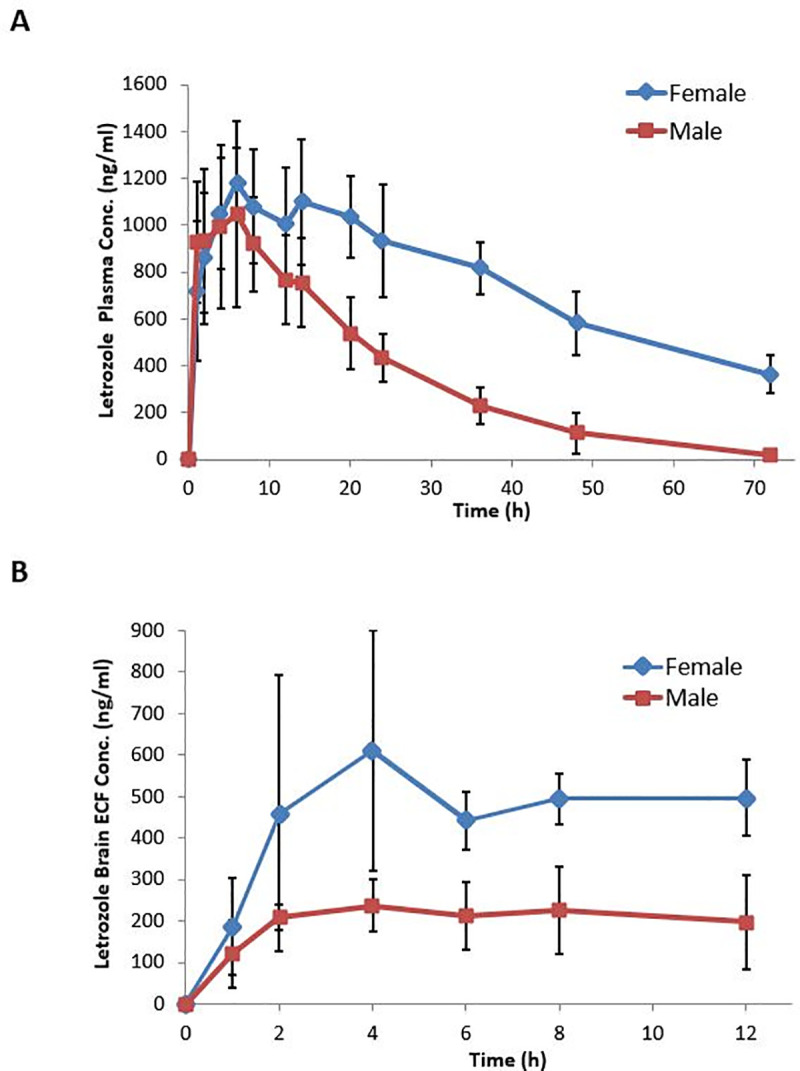
Male versus female letrozole plasma and brain ECF concentration-time profiles in Sprague- Dawley rats. Following single dose administration of letrozole (4 mg/kg), A) Plasma samples were collected through the jugular vein catheters at discrete time intervals from 0–72 hours; B) Microdialysate samples were collected simultaneously with plasma samples up to 12 hours. Dialysate concentrations were corrected for *in vitro* recovery of 7.2%. Data; mean ± SD (N = 3–4 rats/group); mean concentrations of letrozole in females (**♦**) and males (**■**).

Plasma and brain ECF samples were collected simultaneously for 12 hours following the administration of letrozole (4 mg/kg, i.p.). Collection of plasma samples was continued up to 72 hours. Brain ECF concentrations were additionally corrected for the *in vitro* recovery of 7.2%, which is consistent with our previous *in vivo* recovery of 9.5% [14]. The plasma concentration- time profiles of letrozole obtained in both male and female rats are illustrated in Fig 2A. Various parameters estimates such as Cmax, Tmax, t1/2 and AUC0- 72 were calculated for each group using NCA and are presented in Table 2. As shown, we observed marked gender-based differences in the single dose letrozole pharmacokinetics in rats. The systemic clearance in female rats was significantly lower than that in male rats (57.4 ± 13.0 ml/h/kg vs 145.9 ± 14.0 ml/h/kg; p = 0.0003). The AUC0-72 attained in female rats was about 2.0-fold higher (54,390 ± 13100 h*ng/ml) relative to that in male rats (24,860 ± 5040 h*ng/ml) (p = 0.0052). The mean Cmax values were, however, not significantly different in the two groups (1,200 ± 160 vs 1,060 ± 360 ng/ml; p = 0.582). Consistent with the overall change in the systemic clearance, the terminal half-life was substantially higher in female rats (34.0 ± 3.6 h vs 9.2 ± 2.4 h in males; p = 0.0004), while the mean Tmax values were comparable in the two groups (8.5 vs 6.5 h; p = 0.39).

**Table 2:** Brain and plasma pharmacokinetics and unbound partition coefficients (Kp_uu_) of letrozole after a single dose of 4 mg/kg in both male and female Sprague-Dawley rats.

PK parameter	Brain ECF	Plasma_total_	Plasma_ub_^*a*^	Kp_uu_^*b*^
***Males***				
N	3	4		
Tmax (h)	4.7 (3.6)	6.5 (1)	-	
Half- life -t_1/2_ (h)	-	9.2 (2.4)	-	
Cmax (ng/ml)	260 (80)	1060 (360)	403 (136)	0.6
AUC _0–12_ (h.ng/ml)	2410 (1100)	10700 (3000)	4066 (1140)	0.6
AUC _0–72_ (h.ng/ml)	-	24860 (5040)	9447 (1920)	
CL (ml/h/kg)	-	146 (14.0)	-	
***Females***				
N	4	4		
Tmax (h)	6.9 (5.4)	8.5 (5.2)	-	
Half- life -t_1/2_ (h)	-	34.0 (3.6)***	-	
Cmax (ng/ml)	660 (260)*	1200 (160)	456 (60)	1.4
AUC _0–12_ (h.ng/ml)	5750 (2160)*	11700 (3060)	4446 (1160)	1.3
AUC _0–72_ (h.ng/ml)	-	54390 (13100)**	20668 (4980)	
CL (ml/h/kg)	-	57.4 (13.0)***	-	

Observed data presented as mean (SD). Significance was determined for the male versus female groups (*p < 0.05, **p < 0.01, and ***p < 0.001).

^*a*^Plasma_ub_ is the concentration of letrozole in plasma not bound to plasma proteins (fraction unbound for letrozole is 0.38).

^*b*^Kp_uu_, partition coefficient in brain, measured as the ratio of C_max_ or AUC_0-12_ of letrozole in brain ECF to the corresponding values in plasma when corrected for protein binding.

The single dose pharmacokinetic profiles of letrozole in male and female brain ECF are shown in Fig 2B which depict similar gender-based differences in the CNS exposure of the drug. As illustrated in Table 2, ECF AUC0-12 values for the female and male rats were 5,750 ± 2160 and 2,410 ± 1100 h*ng/ml, respectively, representing a 2.4-fold difference (p = 0.048). Correspondingly, the mean ECF Cmax values were 660 ± 260 and 260 ± 80 ng/ml demonstrating a 2.5-fold difference (p = 0.046). However, the rate of letrozole penetration across the BBB, as gleaned from the brain ECF Tmax, was not statistically significant between the two groups, consistent with the single dose plasma data (Tmax of 6.9 h vs 4.7 h in males; p = 0.22).

### Steady state pharmacokinetics and accumulation of letrozole in male and female rats

**Fig 3:**
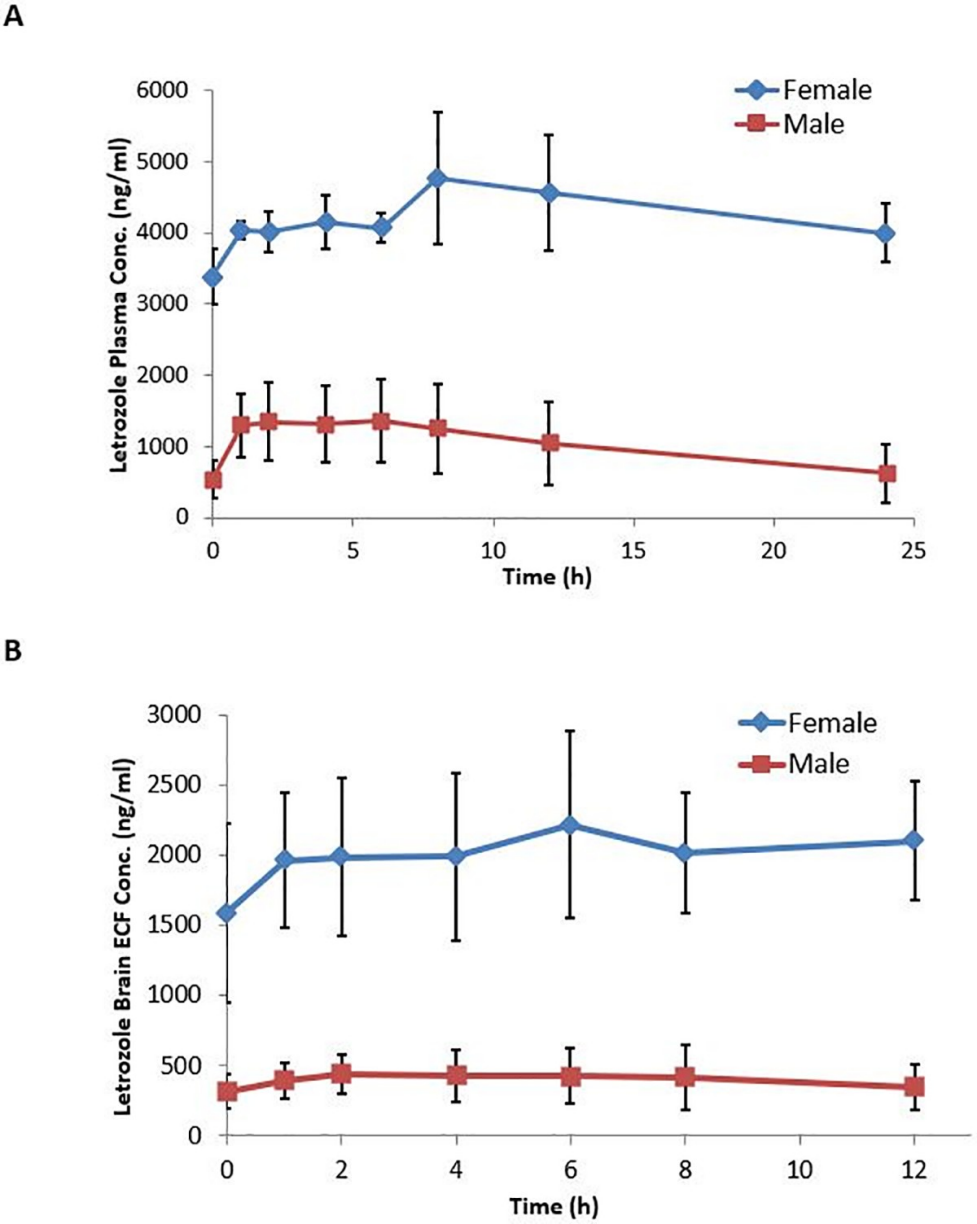
Steady state plasma and brain ECF pharmacokinetics of letrozole in male and female Sprague- Dawley rats. Letrozole (4 mg/kg) was given once daily for 5 or 11 days to male and female rats respectively to establish steady state. A) Plasma samples were collected via the jugular vein catheters up to 24 hours following drug administration. B) Simultaneous microdialysate sample collection was carried out from 0–12 hours. Dialysate concentrations were corrected for *in vitro* recovery of 7.2%. Concentration–time plots were obtained and the data were presented as mean ± SD (N = 4–6 rats/group); mean concentrations of letrozole in females (**♦**) and males (**■**).

To compare gender-specific systemic and CNS pharmacokinetic differences after steady state dosing, letrozole (4 mg/kg, i.p.) was administered daily for 5 and 11 days to male and female rats respectively, prior to conducting blood and ECF sampling. The plasma concentration- time profiles at steady state are shown in Fig 3A and the pertinent steady state pharmacokinetic parameters are listed in Table 3. At steady state, the AUC and Cmax differences between the two genders, noted following single dose administration, were further magnified. The AUC0-24 in female rats was about 4.0-fold higher than that in male rats (102,970 ± 13860 vs 24,800 ± 12260 h*ng/ml) which was highly statistically significant (p = 0.0001). Unlike our observation with single dose administration, differences in the Cmax were observed at steady state. Accordingly, the Cmax was about 3.0-fold higher (4,830 ± 946 vs 1,490 ± 550 ng/ml) (p = 0.0001) in female rats. While the systemic clearance of letrozole was not statistically different following single dose and steady state dosing in male rats, the clearance was lower in female rats at steady state (39.3 ml/h/kg) and this reduction was deemed statistically significant (p = 0.036). As such, the overall difference in the systemic clearance of letrozole at steady state between male and female rats was approximately 5.0-fold.

**Table 3:** Steady state brain and plasma pharmacokinetics, accumulation indices and unbound partition coefficients (Kp_uu)_ of letrozole in both male and female Sprague-Dawley rats.

PK parameter	Brain ECF	Plasma_total_	Plasma_ub_^*a*^	Kp_uu_^*b*^	R_ac brain_^*c*^	R_ac plasma_^*c*^
***Males***						
N	6	6				
Tmax (h)	3.3 (3.8)	3.3 (2.9)	-			
Cmax (ng/ml)	490 (240)	1490 (550)	566 (209)	0.9	1.9	1.4
AUC _0–12_ (h.ng/ml)	4820 (2560)	14800 (6400)	5624 (2430)	0.9	2.0	1.4 (R_ac_’; 1.2)
AUC _0–24_ (h.ng/ml)	-	24800 (12260)	9424 (4660)			
CL (ml/h/kg)	-	200.6 (98)	-			
***Females***						
N	6	4				
Tmax (h)	6.5 (4.6)	5.3 (3.7)	-			
Cmax (ng/ml)	2290 (624)***	4830 (946)***	1835 (359)	1.2	3.5	4.0
AUC _0–12_ (h.ng/ml)	23990 (5660)***	49300 (6210)***	18734 (2360)	1.3	4.2	4.2 (R_ac_’; 2.6)
AUC _0–24_ (h.ng/ml)	-	102970 (13860)***	39129 (5267)			
CL (ml/h/kg)	-	39.3 (5.5)*	-			

Observed data presented as mean (SD). Significance was determined for the male versus female groups (*p < 0.05, **p < 0.01, and ***p < 0.001).

^*a*^Plasma_ub_ is the concentration of letrozole in plasma not bound to plasma proteins (fraction unbound for letrozole is 0.38).

^*b*^Kp_uu_, partition coefficient in brain, measured as the ratio of C_max_ or AUC_0-12_ of letrozole in brain ECF to the corresponding values in plasma when corrected for protein binding.

^*c*^Observed accumulation ratios calculated using Eq (1).

The gender-dependent differences were similarly reflected in brain ECF parameters at steady state. As shown in Fig 3B and Table 3, the steady state Cmax values were 2,290 ± 624 ng/ml and 490 ± 240 ng/ml (p = 0.0006), while the AUC0-12 values were 23,990 ± 5660 h*ng/ml and 4,820 ± 2560 h*ng/ml (p = 0.0002) in females and males, respectively, underscoring an overall 4.7 to 5–fold higher exposure in female rats. For a quantitative evaluation of the letrozole penetration across the BBB *in vivo*, simultaneous plasma and ECF sample collection facilitated estimates of brain-to-plasma unbound partition coefficients (Kpuu). To assure consistent patency of the microdialysis probes, ECF samples were collected only up to 12 hrs. Therefore, the Kpuu values were computed as the ratio of Cmax or AUC0-12 in ECF versus plasma levels corrected for protein binding. Tables 2 and 3 illustrate these ratios at single dose and steady state in both the genders. The partition coefficients ranged from 0.6–0.9 and 1.2–1.4 for the male and female rats respectively indicating facile access of letrozole to the brain.

The extent of drug accumulation at steady state was computed from the ratio of Cmax or AUC0-12 at steady state and corresponding single dose values. A similar pattern of gender-specific differences was noted in both plasma and brain ECF exposure. In males, these ratios indicated approximately 1.4-fold accumulation at steady state, which is consistent with the predicted value. In female rats, however, the extent of accumulation was 4-fold which is considerably higher than the predicted value of 2.6-fold (Table 3).

### PBPK modeling and simulation

**Fig 4:**
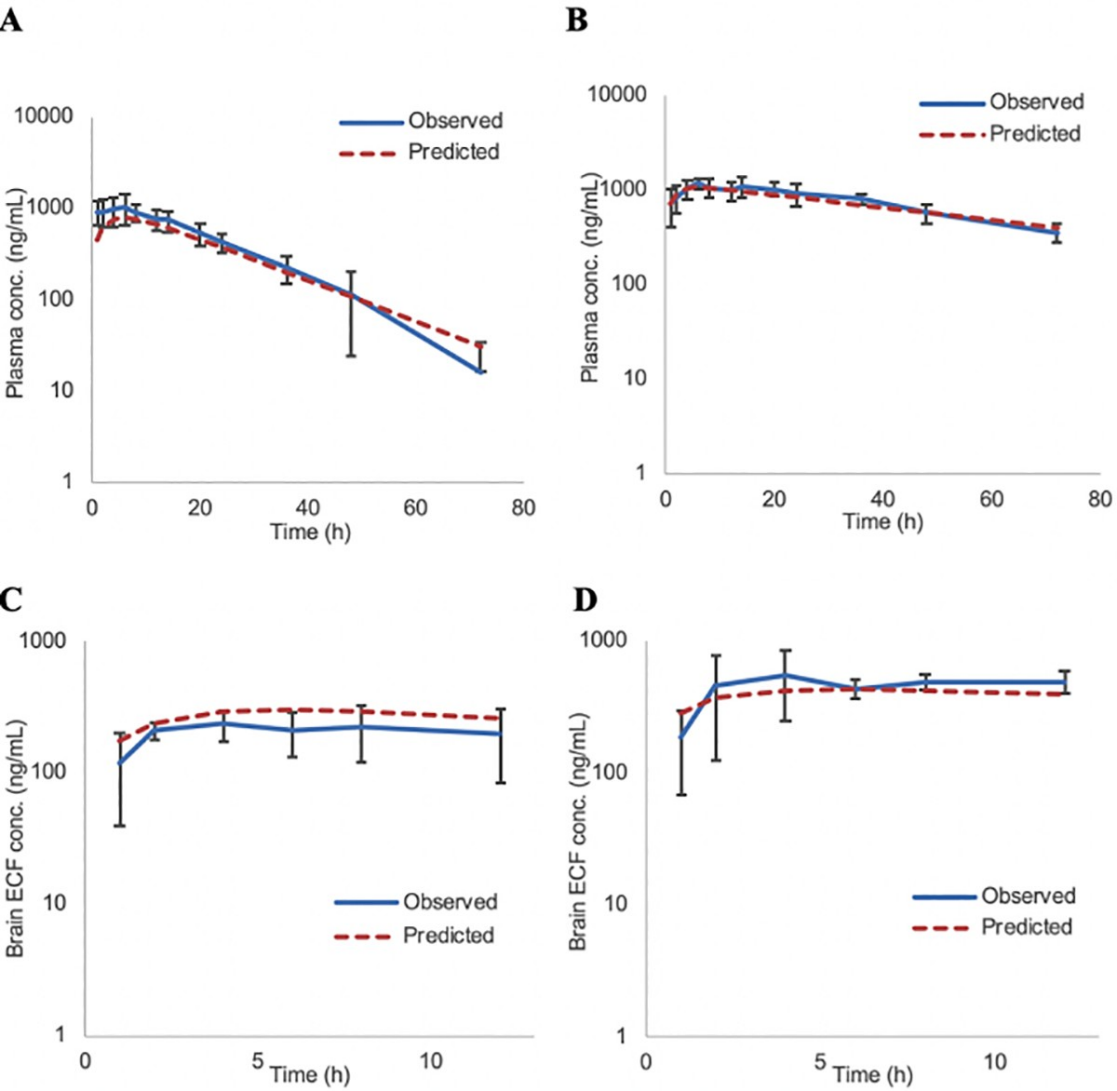
Comparison between the observed vs PBPK model-predicted plasma/ brain ECF concentrations of letrozole in male (A, C) and female (B, D) rats. The brain tissue concentrations were simulated following a single dose of 4 mg/kg using the multi-compartment model as implemented in the Simcyp v17 animal simulator and the ECF concentrations were estimated using f_u_, _brain_ of 0.58. Observed data; mean ± SD; the solid and dashed lines represent overall mean concentration–time profiles for the observed and predicted data respectively.

**Fig 5:**
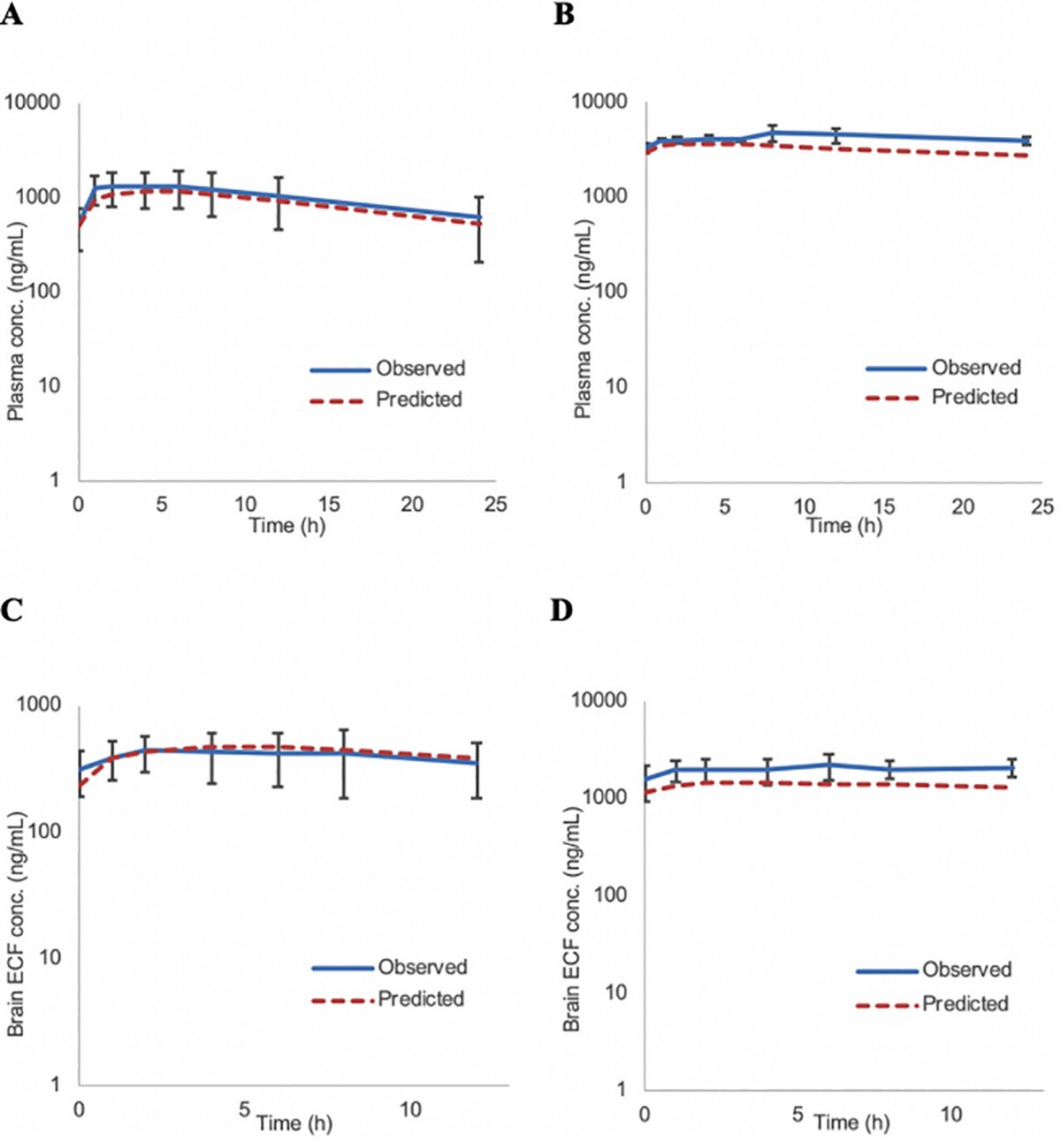
Model-predicted and observed plasma/ brain ECF concentration- time profiles of letrozole at steady state in male (A, C) and female (B, D) rats obtained employing the Simcyp v17 animal simulator. Simulations were performed in virtual rodent population following extravascular dosing of 4 mg/kg of letrozole for 5 and 11 days in male and female rats respectively. Brain tissue concentrations were additionally corrected for f_u_, _brain_ of 0.58 to acquire the brain ECF concentrations. Observed data; mean ± SD; the solid and dashed lines represent overall mean concentration–time profiles for the observed and predicted data respectively.

Simcyp predicted versus the observed concentrations for both male and female rats following a single dose of letrozole (4 mg/kg) are illustrated in Fig 4. As indicated, the simulated concentration-time profiles were comparable with the observed results. Fig 5 presents the predicted steady-state exposure in rats from 0–24 (plasma) and 0–12 (brain ECF) hrs after once-daily administration of 4 mg/kg letrozole for 5 (males) or 11 (females) consecutive days. All the Cmax and AUC predictions for male and female rats met the predefined criterion of 1.5–fold, ranging from 1.04 to 1.5–fold of the observed values. Additionally, simulated data from 8 of the 10 (80% for single dose; Table 4) and 7 of the 8 (88% at steady state; Table 5) predictions were at or within a more stringent 1.3–fold range of those observed in our pre- clinical study. As such, the PBPK model predicted parameters were in good agreement with the observed values.

**Table 4:** Male versus female observed and predicted pharmacokinetic parameter estimates in both brain and plasma of Sprague-Dawley rats following administration of a single dose of letrozole (4 mg/kg) obtained by NCA.

PK parameter	*Males*			*Females*		
	Observed	Predicted	Predicted/observed ratios	Observed	Predicted	Predicted/observed ratios
*Plasma*						
Half- life -t_1/2_ (h)	9.23	13.34	1.45	34.00	43.77	1.29
Cmax (ng/ml)	1060	790	0.75	1200	1080	0.90
AUC _0–72_ (h.ng/ml)	24860	21050	0.85	54390	51870	0.95
*Brain ECF*						
Cmax (ng/ml)	260	310	1.19	660	470	0.71
AUC _0–12_ (h.ng/ml)	2410	3120	1.29	5750	4640	0.81

**Table 5:** Observed versus predicted steady state brain and plasma pharmacokinetic parameters of letrozole in both male and female rats following administration of 4 mg/kg dose for 5 (males) or 11 (females) consecutive days derived by NCA.

PK parameter	*Males*			*Females*		
	Observed	Predicted	Predicted/observed ratios	Observed	Predicted	Predicted/observed ratios
*Plasma*						
Cmax (ng/ml)	1490	1190	0.80	4830	3650	0.76
AUC _0–24_ (h.ng/ml)	24800	21640	0.87	102970	79310	0.77
*Brain ECF*						
Cmax (ng/ml)	490	470	0.96	2290	1510	0.66
AUC _0–12_ (h.ng/ml)	4820	5140	1.07	23990	18730	0.78

## Discussion

Based on previously reported observations that the enzyme aromatase (CYP19A1) is markedly elevated in HGG surgical samples from patients [7, 9] and that the aromatase inhibitor letrozole is effective against malignant glioma in experimental models [8, 31], efforts are in progress to comprehensively characterize the anti-HGG activity of this agent in various animal models. Given that a majority of potentially effective chemotherapeutic agents have limited penetration across the BBB, comprehensive pharmacokinetic studies that provide quantitative insights into the CNS distribution of this agent in both male and female animals are warranted for optimization of pre-clinical efficacy studies. Previously, we have shown that in female Sprague-Dawley rats letrozole easily crosses the BBB following single dose intravenous injections [14]. Here, we have considerably extended these findings to assess single dose and steady state plasma and brain ECF pharmacokinetics of letrozole in both male and female rats.

This comprehensive characterization was partially prompted by an earlier report of gender-dependent changes in the plasma pharmacokinetics of letrozole in rats following single dose administration via oral gavage [16]. Consistent with this report, in our studies letrozole plasma half-life was much longer in female rats (~ 34 hrs) compared to that in male rats (~ 9 hrs). The overall AUC0-72 following single dose administration was 2.0-fold higher in female rats. While the plasma clearance and elimination half-life of letrozole were strikingly different, the Tmax values were comparable. This suggested that the observed variance in the plasma pharmacokinetics of letrozole results from differences in the elimination pathways and not in the rate of drug absorption. Letrozole elimination primarily entails hydroxylation to a carbinol metabolite [32]. A previous study investigated rat liver microsomal metabolism of letrozole and noted that the overall intrinsic clearance and the formation rate of the carbinol metabolite was about 3.0-fold higher in male rats [15].

In humans, letrozole hydroxylation is mainly catalyzed by CYP2A6 with a minor contribution by CYP3A4 [33, 34]. While gender/hormonal effects on the expression of CYP2A6 in humans are not noted [35], the expression of the rat ortholog, CYP2A2, exhibits gender-dependent expression. In male rats CYP2A2 expression is reported to be higher and inducible relative to that in female rats [36]. This sexual dimorphism may be a result of episodic burst of growth hormone in male rats [37]. Indeed, similar sex-dependent pharmacokinetic differences are also noted for other CYP2A2 substrates such as AMG 900 in rats [38].

The gender-specific differences in the systemic exposure of letrozole were further magnified at steady state. The plasma Cmax and AUC0-24 values were 3.0 and 4.0-fold higher in female rats than that in male rats, respectively. The extent of the observed drug accumulation at steady state as reflected in the plasma Cmax and AUC0-12 values was approximately 1.4-fold in male rats, which is consistent with the predicted value (Eq 2). However, these steady state values were about 4.0-fold in female rats, which is considerably higher than the predicted 2.6-fold accumulation. In humans, letrozole exhibits non-linearity in its pharmacokinetics at higher doses, especially following multiple dose administration [39, 40]. Our previous study also documents non-linear pharmacokinetics of letrozole in female Sprague-Dawley rats above single doses of 8 mg/kg [14]. Given that the intrinsic clearance of letrozole in female rats is considerably lower than that in male rats [15], presumably due to differences in the metabolic capacity, letrozole exposure may increase non- linearly at steady state due to saturation of the hepatic metabolism.

Most essentially, our study documents a marked difference in the ECF pharmacokinetics between male and female rats. We employed microdialysis which facilities simultaneous brain ECF and peripheral blood sampling [41], enabling temporal resolution and quantitative estimation of letrozole penetration across BBB and disposition in the brain. The aforementioned differences in the plasma Cmax and AUC are also reflected in the brain ECF data following single dose and steady state drug administration. As such, the brain ECF Cmax and AUC0-12 values in males reflect a 2.0-fold accumulation and 3.5 to 4.0-fold accumulation in female rats. A limitation of the microdialysis was that ECF sampling could be done only for 12 hours post letrozole administration due to chances of reduced patency of the microdialysis probes, which may result in reduced drug recovery at later time points. Thus, the Kpuu (brainecf/plasmaub) values were computed using Cmax and AUC0-12. The Kpuu values after single and steady state dosing were higher in females (1.2–1.4) compared to that in males (0.6–0.9) as reflected in higher brain ECF Cmax values in female rats. The reasons for this apparent difference are not clear but it appears that in addition to the difference in the systemic clearance pathways, the BBB penetration of letrozole in female rats may be higher. The experimental constraint that necessitated determination of brain ECF AUC0-12 is unlikely to contribute to the observed difference as both the AUC0-12 derived Kp_uu_ values are consistent with those observed using C_max_ values. A noteworthy point here is that following multiple dosing letrozole ECF levels fluctuated minimally, especially in female rats, which is consistent with the observed long elimination half-life of the drug. The predicted steady state average concentrations for ECF would be in the range of 210 and 1,000 ng/ml for male and female rats, respectively. These pharmacokinetic observations along with the *in vitro* and *in vivo* efficacy data will provide useful insights for optimizing dosing regimens in further pre-clinical anti-tumor studies. In a recently completed study, we have noted that letrozole half-life in the NSG mice is extremely short (~ 3 hrs) [42]. For complex patient derived xenograft studies, immunocompromised mouse model is usually preferred. However, given the short half-life of letrozole in mice, such testing may require the use of immunocompromised rats. Therefore, further anti-tumor efficacy assessments in these pre-clinical animal models would need to consider the observed gender- dependent differences for designing anti-tumor efficacy studies.

Additionally, we investigated the use of a bottom-up whole body PBPK modeling approach that integrates physico-chemical/ biopharmaceutical properties and plasma pharmacokinetics of the drug and species-specific physiological factors [43], likely to impact drug disposition to the brain to predict the penetration and brain pharmacokinetics of letrozole. The multi-compartment brain model, as specified within the Simcyp® Rat simulator, was employed for this purpose. The physico- chemical (molecular weight, LogP etc.) and biopharmaceutical properties (fraction absorbed, bioavailability etc.) were gleaned from literature. Various pharmacokinetic properties for male and female rats (systemic clearance and brain tissue binding) were experimentally determined in this study. The anatomical and physiological details of the brain and CSF, such as blood flow to the brain; CSF secretion, circulation and absorption were provided in the Simcyp simulator. Letrozole diffusion across the BBB was assumed to be via passive diffusion with no involvement of uptake/efflux transporter based on prior publications [11]. The metabolic clearance of letrozole in the brain was assumed to be negligible. Of note, we could not generate 5th/95th percentile confidence intervals typically observed with simulations conducted to predict pharmacokinetic profile in humans. In the given animal species, determination of inter-subject variability in pharmacokinetic parameters that would allow for constructing such confidence intervals was not feasible. Additionally, the PBPK model facilitated prediction of brain tissue concentrations of letrozole not the ECF levels. Therefore, to derive the simulated brain ECF letrozole pharmacokinetic profile, we first simulated the brain tissue concentrations and corrected those for the fraction unbound in the brain mass (fu, brain) since the unbound fraction of the drug in the brain tissue is in equilibrium with the ECF drug levels. This approach is consistent with other previously published studies such as that of [44] where unbound drug concentration in the brain mass served as a surrogate for the brain ECF concentration. While we did not perform a comprehensive sensitivity analysis, the PBPK modeling derived plasma and brain ECF concentrations were most sensitive to the absorption rate constant (K_a_) and oral clearance (CL_po_) obtained from the previously published [16]. The slight over prediction of the plasma and brain ECF levels in female rats may be a result of somewhat higher clearance observed in our study (~ 0.25 ml/min) relative to that reported earlier (0.21 ml/min). Overall, the PBPK model-predicted brain ECF concentration profiles of letrozole compared well with the observed profiles. Indeed, all the letrozole exposure predictions were within 1.5-fold of the observed values.

In summary, our study represents a comprehensive assessment employing pre-clinical studies to determine the extent of letrozole accumulation and penetration across the BBB. It highlights the important gender-based differences in the systemic elimination profile and the net plasma and brain ECF pharmacokinetics of letrozole in rats following both single dose and at steady state. The Kpuu values for letrozole suggests robust penetration across BBB in both male and female rats and the observed steady state brain ECF letrozole concentrations will serve as a guide for dosing strategies in subsequent pre- clinical efficacy studies. While, letrozole is currently approved for breast cancer treatment and as such it is primarily used in the post-menopausal women, plasma pharmacokinetics of this drug were investigated in both genders during its early clinical development stages. No gender-dependent differences in the systemic pharmacokinetics were apparent in studies that enrolled healthy volunteers (CDER NDA 20–726). This is consistent with the observed lack of sexual dimorphism in the expression of CYP2A6 expression in humans [35]. Thus, the observed gender-based variability in letrozole pharmacokinetics in rats may not be of clinical concern. Nonetheless, our observations are important for pre-clinical pharmacology and toxicology assessments of letrozole. The dose we employed here is similar to the 2 mg/Kg reported by Liu et.al. 2000 from where we derived the key parameters for PBPK modeling [16]. The observed steady state brain ECF concentrations of letrozole in male and female rats exceed the cytotoxic concentrations of letrozole observed against patient-derived glioblastoma cell lines. In a previous study we observed marked reduction in tumor volume in the rats with orthotopic implantation of C6 glioma tumor cells at letrozole dose employed here (4 mg/kg) [8]. Thus, these data are important to provide additional guidance for pre-clinical development of letrozole as a new therapeutic for brain tumors. An important aspect of our study was the development of a quantitative PBPK model that may serve as a good predictive tool for brain pharmacokinetics of letrozole. With further refinement of the model with human-specific pharmacokinetic parameters, the model has the potential to serve as an important translational tool for predicting human brain drug levels.
